# The Role of Nanomaterials in Modulating the Structure and Function of Biomimetic Catalysts

**DOI:** 10.3389/fchem.2020.00764

**Published:** 2020-09-29

**Authors:** Yanyan Huang, Deshuai Yu, Yibin Qiu, Lanlin Chu, Youhui Lin

**Affiliations:** ^1^College of Light Industry and Food Engineering, Nanjing Forestry University, Nanjing, China; ^2^Fujian Provincial Key Laboratory for Soft Functional Materials Research, Department of Physics, Research Institute for Biomimetics and Soft Matter, Xiamen University, Xiamen, China

**Keywords:** enzyme mimic, structure and function, catalysis, nanomaterial, natural enzyme

## Abstract

Nanomaterial-incorporated enzyme mimics have so far been examined in various cases, and their properties are governed by the properties of both catalysts and materials. This review summarizes recent efforts in understanding the role of inorganic nanomaterials for modulating biomimetic catalytic performance. Firstly, the importance of enzyme mimics, and the necessity for tuning their catalysis will be outlined. Based on structural characteristics, these catalysts are divided into two types: traditional artificial enzymes, and novel nanomaterial-based enzyme mimics. Secondly, the mechanisms on how nano-sized materials interact with these catalysts will be examined. Intriguingly, incorporating various nanomaterials into biomimetic catalysts may provide a convenient and highly efficient method for the modulation of activities as well as stabilities or introduce new and attractive features. Finally, the perspectives of the main challenges and future opportunities in the areas of nanomaterial-incorporated biomimetic catalysis will be discussed. In this regard, nanomaterials as a kind of promising scaffold for tuning catalysis will attract more and more attention and be practically applied in numerous fields.

## Introduction

Owing to their excellent catalytic efficiency, unique mechanistic pathways, and complicated structural features, natural enzymes have become a tremendous source of inspiration for chemists. Numerous studies have concentrated on the simulation of their structural characteristics and functions (Hooley, 2016). So far, a variety of synthetic structures, such as porphyrins, cyclodextrins, organoselenium, and metal complexes, have been widely explored to design and construct artificial enzymes through various approaches (Dong et al., 2012; Du et al., 2017; Elemans and Nolte, 2019). In recent years, with the development of nanotechnology, many functional nanomaterials have emerged. Carbon-based, silicon-based, and metal-based nanomaterials are often endowed with properties such as large specific surface areas, easy surface modification, and high recycling efficiency. The rapid development of nanotechnology and biology has provided new opportunities for the construction of different nano-scaled structures with enzyme-like catalytic properties (Jiang et al., 2019; Liang and Yan, 2019; Wu et al., 2019). With the assistance of nanomaterials, active molecules or nanoparticles can be well-dispersed. Furthermore, bi- or multi-active components can be assembled in one nano-scaled system successfully. As a new generation of artificial enzymes, such nanocatalysts, including Fe_3_O_4_ (MNPs) (Gao et al., 2007), CeO_2_ (Xu and Qu, 2014), V_2_O_5_ (Ghosh et al., 2018), AuNPs (Gao et al., 2015), MoS_2_ nanosheets (Yin et al., 2016), graphene oxide (GO) and few-layer grapheme (Song et al., 2010), and other types of nanoparticles (Singh et al., 2017) are particularly impressive. Note: although the assembly of active components such as metal complexes and porphyrins on nanomaterial-based supports leads to the generation of nanocomposites (Wang et al., 2013; Huang Y. Y. et al., 2017), these examples treated in this review are categorized into traditional artificial enzymes, instead of nanomaterials-based artificial enzymes. The synthetic biocatalysts often possess the properties such as low cost, easy preparation, and anti-biodegradation as well as anti-denaturation when compared with natural enzymes (Huang et al., 2019). Although promising, the inherent limitations of the native forms hamper their practical applications. It is worth noting that these mimics often have relatively low catalytic performances. In this way, they can't match the high catalytic activities of natural enzymes. Furthermore, it is highly desirable that the generated enzyme mimics not only simply duplicate and mimic natural enzymes' inherent characteristics, but also may exhibit further novel properties for biological applications. Based on these unique and attractive features mentioned above, nanomaterials may also provide excellent scaffolds to the development of biomimetic catalysis for potential applications (Fan et al., 2018; Han et al., 2019, 2020).

**Figure 1:**
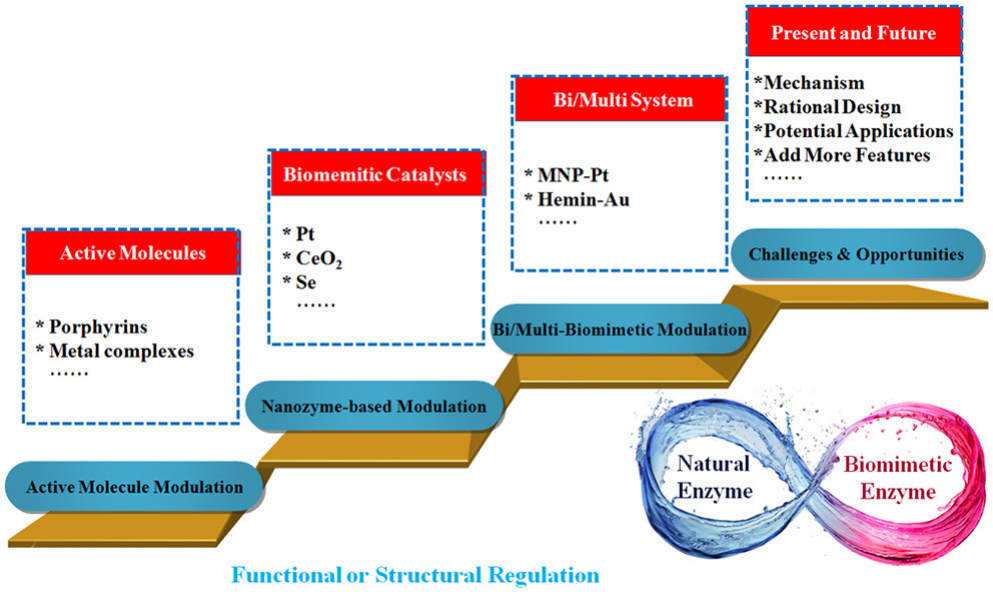
Scheme describing the incorporation of nanomaterials into artificial enzymes for tuning catalysis.

Recently, numerous efforts have been devoted to exploring the behavior of nanomaterials in biomimetic systems (Wang et al., 2019). This review aims to provide an overview on recent developments in tuning biomimetic catalysis using nanoscale inorganic materials (Figure 1). The incorporation of nanomaterials into biomimetic catalysts provides a convenient and highly efficient method for the modulation of activities as well as the stabilities of catalysts. Alternatively, this can introduce unique and attractive features, which are not possessed by the catalysts themselves. The biomimetic catalysts discussed in this review can be roughly divided into two types according to their structural characteristics, which are traditional artificial enzymes and nanomaterials-based artificial enzymes. We hope this review may accelerate further progress in these promising areas.

## Traditional Artificial Enzymes Modulation

One challenge in chemistry has been the construction of synthetic systems that mimic the functions of natural enzymes. Until now, the artificial enzyme field has achieved remarkable progress. Furthermore, the incorporation of nanomaterials into these mimics have great potential for tailoring their catalytic activities and introducing other attractive features. This section will discuss nanomaterials when they interact with active molecules with enzyme-like properties.

### Tuning Catalysis of Active Molecules

#### Nanomaterials for Minimizing Dimerization and Oxidative Degradation

Metal-organic-frameworks (MOFs) and their derivatives have served as outstanding supports for heterogeneous catalysis owing to their unique features such as large specific surface areas, excellent electron transfer ability as well as rich surface chemistry. Inspired by this, a study demonstrated that metalloporphyrins could be successfully assembled with secondary binding units of metal clusters to form 2D bimetallic MOF nanosheets (Wang et al., 2016). It is well-known that porphyrin molecules easily form dimers, which may affect their catalytic activity. After self-assembling, the obtained nanomaterials could efficiently disperse the metalloporphyrins and achieve an excellent biomimetic property to catalyze the co-oxidation reaction. Based on this work, Zhang et al. further designed Au-modified metalloporphyrinic MOF nanosheets. The obtained nanocomposites could efficiently disperse both Au nanoparticles and metalloporphyrin molecules. In this way, the nanocomposite could serve as a biomimetic catalyst for glucose-H_2_O_2_ cascade reaction (Huang Y. Y. et al., 2017).

#### Nanomaterials for Increasing Binding Affinity to Substrates

Natural enzymes have extraordinarily high catalytic efficiency. This is largely owing to their ability to bring corresponding substrates close to their active sites. Zhang et al. found that graphene oxide (GO) could dramatically increase the nuclease-like activity of the copper complexes-based DNA intercalators (Zheng et al., 2012). In their system, the copper complexes could be modified onto the surface of GO *via* π-π interaction. The nuclease activity of the resulting conjugates was significantly higher than that of the copper complexes alone. One reason for the DNA cleavage enhancement by GO is that the obtained conjugates have a much higher binding affinity to the DNA molecules. Furthermore, another reason may be related to the generation of reactive species by accelerating the reduction of the metal center (Zheng et al., 2014).

#### Nanomaterials for Constructing Multivalent Catalysts

A previous review on catalysis by colloid aggregates mentioned “…groups of molecules, properly assembled, can obviously accomplish much more than an equal number of molecules functioning separately” (Menger, 1991). As the evidence began to accumulate that a lot of biological systems function *via* the simultaneous effects of multiple interactions, this observation was becoming increasingly important. To date, significant progress in self-assembly of catalytic-active monolayers on multivalent scaffolds, such as micelles (Dong et al., 2012), liposomes, proteins (Hou et al., 2012), and nanoparticles, has been achieved.

**Figure 2:**
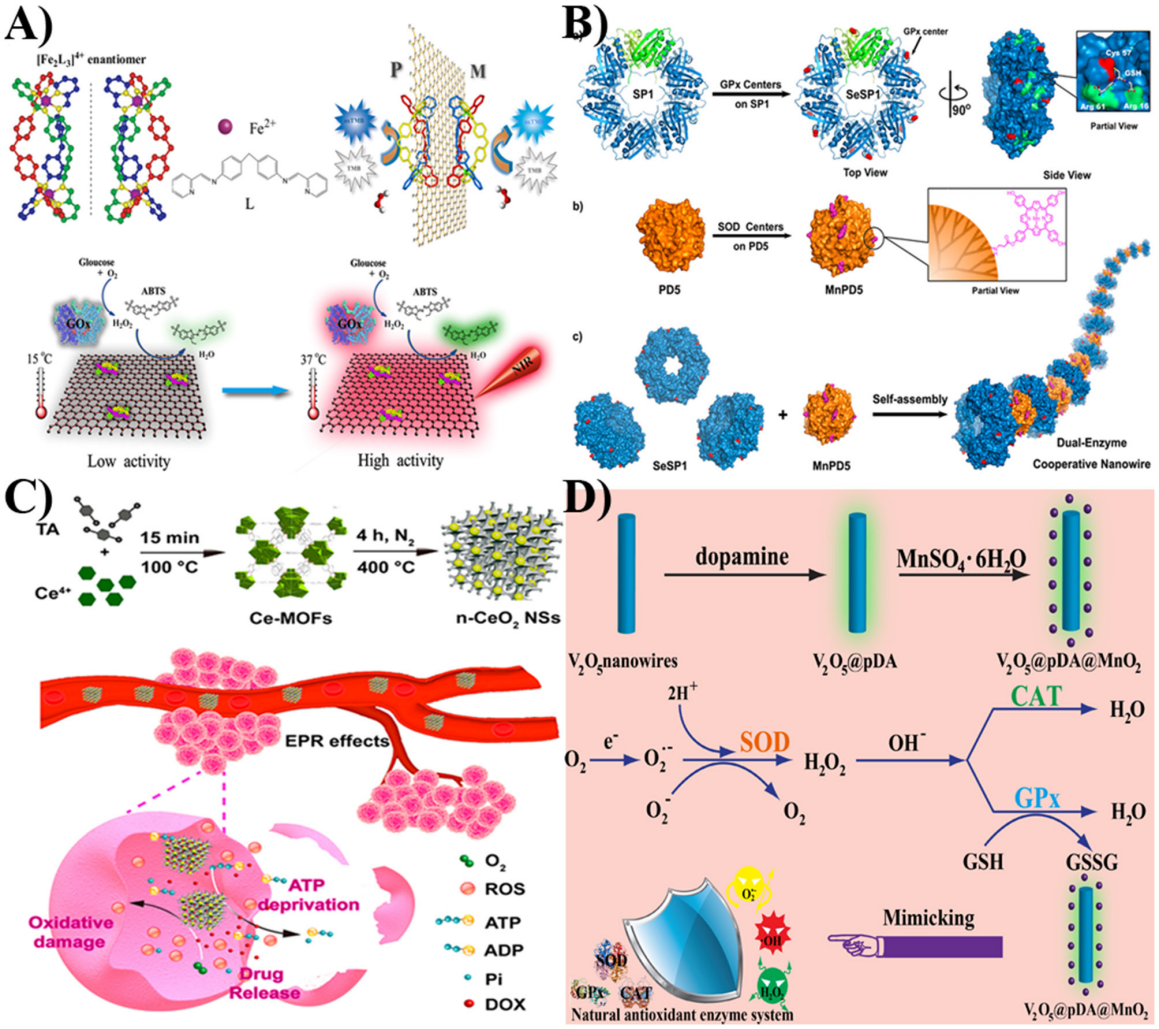
**(A)** The peroxidase-like property of [Fe_2_L_3_]^4+^/GO-COOH can be controlled by NIR. Reprinted with permission from Xu et al. Copyright (2014) Wiley-VCH. **(B)** The assembly of SeSP1 and MnPD5 to form dual-enzyme cooperative nanowire. Reprinted with permission from Sun et al. Copyright (2015) American Chemical Society. **(C)** The design of the CeO_2_ NPs encapsulated-porous carbonaceous frameworks for cancer therapy. Reprinted with permission from Cao et al. Copyright (2018) American Chemical Society. **(D)** The assembly and the scheme of the V_2_O_5_@pDA@MnO_2_ nanozyme for mimicking natural antioxidant enzyme system. Reprinted with permission from Huang et al. Copyright (2016) Wiley-VCH.

For example, Liu et al. used poly(amido amine) dendrimers (PD5) and cricoid proteins (SP1) as the templates to bind with superoxide dismutase (SOD) and glutathione peroxidase (GPx) catalytic centers, respectively (Figure 2A). The obtained MnPD5 and SeSP1 were then self-assembled to form dual-enzyme cooperative nanowires. This nanocomposite could exhibit excellent SOD- and GPx-like properties to remove harmful reactive oxygen species (ROS) (Sun et al., 2015).

### Introducing Additionally New and Attractive Features

By incorporating functional nanomaterial into enzyme mimics, such biomimetic catalysts cannot only mimic the functions of natural enzymes, but also possess additionally advanced features in some cases.

#### Assembling an Electrode by Binding Active Sites to Nanomaterials

The interconversion of water and hydrogen in unitized regenerative fuel cells is considered as one important energy storage method to eliminate the temporal fluctuations of wind power and solar power. Nevertheless, to enable this technology to be economically viable, replacing current commercial platinum catalysts with cheaper and more abundant materials is highly desirable and challenging (Bashyam and Zelenay, 2006). A competitive alternative can be found in microorganisms which can metabolize molecular hydrogen using hydrogenases. Inspired by this, nanomaterials with a hydrogenase-like property can be modified on the electrodes for catalyzing this interconversion. Artero et al. prepared a noble metal-free catalytic nanomaterial through assembling a nickel bisdiphosphine-based complex and multiwalled carbon nanotubes (MWNTs). The obtained nanocomposites could serve as hydrogenase mimics. As a result, this hybrid could act as a highly specific surface area cathode material with outstanding catalytic performance even in the condition of strong acid solutions (Goff et al., 2009).

#### Near-Infrared Photothermal Control

Qu et al. demonstrated an approach to construct a [Fe_2_L_3_]^4+^/GO-COOH-based peroxidase mimic. Since GO-COOH had strong absorption in near infrared regions, this peroxidase mimic had good sensitivity to NIR and high photothermal conversion efficiency (Figure 2B). Furthermore, the integration of this nanocomposite with glucose oxidase (GOx) could enable the creation of a catalytic ensemble for a cascade reaction. In addition, within a temperature range from 15 to 37°C, GOx and [Fe_2_L_3_]^4+^-GO-COOH had a higher activity at an elevated temperature. In the presence of NIR laser irradiation, an obvious absorbance band at 417 nm was achieved, indicating that the oxidized 2,2′-azino-bis(3-ethylbenzothiazoline-6-sulfonic acid) (ABTS^·+^) was formed. Based on NIR photothermal effect and temperature-dependent activity, the activity of a GOx-[Fe_2_L_3_]^4+^-GO-COOH cascade system could be controlled by near infrared (NIR) light (Xu et al., 2014). Taken together, based on the unique advantages of nanomaterials, they can serve as regulators to modulate the catalytic activities of active molecules with enzyme-like properties.

## Modulation of Nanomaterial-Based Artificial Enzymes

So far, a variety of nano-scaled materials have been discovered to have unique enzyme-like activities (Wu et al., 2019). Additionally, a few “small molecule” systems, such as deoxyribonucleic acid and ionic liquid, have shown the ability to modulate the activity of these novel enzyme mimics. In this section, as an alternative to small molecule inhibition, recent progress in nanomaterials provide a novel pathway to regulate the catalytic behaviors of nanomaterial-based artificial enzymes.

### Regulation of Stability and Activity of Nanomaterials-Based Artificial Enzymes

The rapid advances in solid-supported catalysts prompt scientists to examine whether matrices can promote the catalytic behavior of these nano-sized enzyme mimics. Compared with bulk materials, materials filled in the nanochannels often exhibit superior performances such as enhanced catalytic activities and improved stabilities. Ling and Gao's group constructed combined Fe porphyrin and Zr^4+^ ions within MOFs. The obtained nanomaterials were noted as PMOF(Fe) and further assembled with ultrasmall Pt nanoparticles to generate Pt@PMOF(Fe). The MOFs could efficiently hinder the aggregation of the Pt component. In this way, the nanocomposite exhibited high peroxidase-like property and stability (Ling et al., 2020).

In addition, Qu et al. demonstrated one rational method for constructing CeO_2_ NPs encapsulated on porous carbonaceous frameworks (Figure 2C). The porous carbonaceous frameworks could promote the formation of a high degree of very small, well-dispersed, and stable CeO_2_ NPs. The obtained nanocomposites exhibited excellent oxidase-like ability compared with other types of CeO_2_ NPs (Cao et al., 2018). Recently, they used GO as the template to assemble selenium nanoparticles. The formed GO-Se nanocomposites could serve as GPx mimics to eliminate harmful H_2_O_2_ with the assistance of glutathione. This enzyme mimic performed enhanced enzyme-like activities in comparison to their independent components and exhibited potential antioxidant effect for cytoprotection (Huang Y.Y. et al., 2017). Besides the mentioned materials, a carbon nanotube can also be used as a support for regulating the stability and activity of nanomaterial-based artificial enzymes.

### Forming Hybrid Nanomaterials for Artificial Cascade Systems

In addition to regulating catalysis, nano-sized enzyme mimics can also be used for constructing artificial enzymatic cascade systems. For example, Qu et al. used polydopamine (pDA) as the bridge for assembling V_2_O_5_ nanowires and MnO_2_ nanoparticles (Figure 2D). In their work, the MnO_2_ component could serve as SOD mimics to transform a superoxide radical to H_2_O_2_ and O_2_. With the inherent catalase-like property of MnO_2_ and the GPx-like ability of the V_2_O_5_ component, the generated H_2_O_2_ would be scavenged as harmless products. With the antioxidant enzyme-like properties of nanozymes and the antioxidant ability of pDA, the nanocomposites could effectively scavenge overexpressed ROS and protect intracellular components against oxidative damage. This V_2_O_5_@pDA@MnO_2_ nanozyme could mimic intracellular antioxidant enzyme-based defense systems for cytoprotection. Further animal inflammatory models illustrated that the nanocomposites could serve as potential nanoagents for ameliorating inflammation (Huang et al., 2016). Owing to their advantages of large specific surface area, easy surface modification, and excellent electron transfer ability, nanomaterials have been used as promising regulators to control the catalytic properties of artificial enzymes.

## Modulation of Multiple Bio- or/and Biomimetic Catalysts

The attachment of different bio- or/and biomimetic catalysts on the same carbon nanomaterial provides a simple pathway for fabricating catalytic ensembles which possess synergic and complementary properties. Besides porous silica structures, mesoporous carbon can serve as a support for the construction of nanostructured multi-catalyst systems. For instance, combining MNPs with Pt nanoparticles in ordered mesoporous carbon has been prepared by Park et al. (Kim et al., 2014). As a result, this composite exhibited the enhanced peroxidase-like activity in comparison to independent MNPs, which could be due to the synergetic effect.

In addition to traditional graphene-based (Xue et al., 2014) and porous-based materials (He et al., 2013), graphene-mesoporous silica hybrid as a novel nanomaterial can be used for bio- or multi-artificial catalyst modulation. Very recently, Qu and Ren's group reported that hemin and AuNPs with complementary functions could be located in different regions in a graphene-mesoporous silica hybrid. Firstly, through weak π-π stacking interactions, hemin was tethered on the exposed surface of graphene. Since graphene could hinder the self-dimerization of hemin molecules, the as-prepared hemin-contained nanoconjugates can act as a highly efficient peroxidase mimic. Then, AuNPs, with glucose oxidase-mimicking activity, can be formed on the NH_2_ groups functional silica surface by *in-situ* reduction. Consequently, these integrated catalysts exhibit glucose oxidase-like and peroxidase-like catalytic activities. More importantly, these nanocomplexes with multiple catalytic sites are able to catalyze artificial cascade reactions, without the addition of natural enzymes. This finding might pay the way to anchor multiple enzyme mimics on solid matrices for multicomponent cascade transformation or realizing artificial organelles in the future (Lin et al., 2015). In addition to regulating the activity of active molecules or biomimetic catalysts, nanomaterials can also be used for the regulation of multi-biomimetic systems.

## Challenges and Future Opportunities

Although remarkable progress has been made, the development of nanomaterials incorporating enzymes and artificial enzymes is still in a relatively early stage. In order to carry out further research in these mentioned areas, the following challenges need to be addressed:

1. The mechanism leading to the change of catalytic performance. Obvious changes in activity, specificity, or selectivity of these catalysts may appear when combining artificial enzymes with various nanomaterials. However, in many cases, the mechanism on how nanoscale materials affect the properties of these catalysts is not properly understood and validated.
2. Rational design of surface functionalized nano-sized materials. It is important to take into account surface properties in the interactions of nanomaterials with bio- and biomimetic catalysts.
3. Apart from the surface properties, the size, morphology as well as composition of nanomaterials also play a crucial role. This point needs systematical examination in the future.
4. Constructing novel integrated catalysts which possess superior and often unique functions for their practical applications is still in its infancy. More attention should be devoted to the use of functional nanomaterials for constructing catalysts with new properties.
5. Although many enzymes and artificial enzymes have been combined with nanomaterials, it is indeed necessary to further investigate the potential applications of nanomaterials in tuning other biomimetic reactions.
6. It is important to further investigate their potential industrial applications.

## Conclusions

Enzymes have attracted scientists' curiosity and attention for a long time. The concepts of enzyme-catalyzed transformations have been a tremendous source of inspiration for fabricating synthetic catalysts which possess the ability of mimicking the essential or general properties of natural enzymes. Particularly, recent developments in nanotechnology have enhanced the possibility for assembling engineered nanomaterials with biomimetic catalysts. This review systematically summarized the latest developing progress of nanomaterials especially inorganic nanomaterials in regulating biological and biomimetic catalysis. These works have exhibited great potential for applications ranging from the control and regulation of activity and biosensing to the separation and construction of hybrid nanoarchitectures. Evidentially, breakthroughs in biotechnology, nanotechnology as well as bionic technology may pave the way for constructing novel hybrid structures for broad applications by overcoming the unresolved issues and challenges.

## Author Contributions

YH, DY, YQ, LC, and YL conceived and wrote this paper. All the authors read and approved the final manuscript.

## Conflict of Interest

The authors declare that the research was conducted in the absence of any commercial or financial relationships that could be construed as a potential conflict of interest.
